# Exploring the resilience skills and strategies of social workers

**DOI:** 10.4102/hsag.v30i0.2795

**Published:** 2025-05-30

**Authors:** Lucy R. Mangolele, Taetske M. Calitz

**Affiliations:** 1 Department of Social Work, School of Social Sciences, College of Human Sciences, University of South Africa, Pretoria, South Africa

**Keywords:** burnout, experiences, social worker, skill, strategies, stress, qualitative research, resilience

## Abstract

**Background:**

Research shows that social workers face numerous challenges because of the demanding nature of their profession, necessitating the development of resilience skills and techniques. Social workers assist marginalised populations and operate in challenging environments, requiring strong resilience strategies.

**Aim:**

This article examines the resilience skills and strategies of social workers.

**Setting:**

The study setting comprised the Department of Social Development in Johannesburg Metro Region.

**Methods:**

The study employs a qualitative approach, utilising purposive sampling to identify participants for semi-structured interviews. In-depth individual interviews were conducted with the social workers, and data were analysed using Creswell and Poth’s qualitative thematic analysis strategy. A total of 13 participants were interviewed.

**Results:**

Three main themes emerged: Theme 1: Resilience skills and strategies employed by social workers to surmount challenges; theme 2: Effectiveness of resilience skills and strategies employed by social workers; and theme 3: Social workers’ suggestions on what the department can do to maintain their resilience.

**Conclusion:**

The findings demonstrate that resilience skills and strategies are crucial for social workers, enabling them to cope effectively with work-related challenges and find meaning in their work.

**Contribution:**

This study will assist social workers to understand the skills and strategies that underpin their resilience. It will also assist the Department of Social Development and other public sectors in developing support programmes to retain social workers for the profession.

## Introduction

Resilience has become a prominent theme in contemporary literature (Grant & Kinman 2020:4). Resilience is a complex, context- and culture-sensitive process that involves both individual traits and supportive social environments working together to foster adaptability and strength (Theron 2020). This means that the ability to be resilient is influenced by both personal attributes – such as strengths, agency and coping skills – and the surrounding social systems, which include family, school and community supports. Van Breda (2018) also presents resilience as a complex and multifaceted concept in the context of social work practice, defining it as a multilevel process. This process-oriented perspective highlights resilience not as an individual trait but as something that is influenced by a variety of mediating factors that can facilitate positive outcomes despite challenges (Van Breda 2018). Resilience is defined as the capacity to respond to adversity by ‘bouncing back’ or adapting to such an extent that individuals produce positive outcomes and thrive under challenging conditions (Hurley 2020).

The social work profession faces high emotional demands (Travis, Lizano & Mor Barak 2016) and is known for its challenging and stressful nature (Hardy 2017). Social workers encounter various challenges, including high caseloads, lack of resources, insufficient support from managers and client-related difficulties, all of which negatively impact their well-being (Day, Hartling & Mackie 2015; Engelbrecht 2014:101; Moyane 2016; Truter & Fouché 2019). Despite the emotional toll of their work, social workers often refrain from expressing their emotions at the workplace to avoid appearing incapable of handling their responsibilities (Grant, Kinman & Alexander 2014).

Developing emotional resilience is essential to retain social workers in their careers, prevent burnout, enabling them to cope with stress and bounce back from challenges and to perform their roles efficiently and effectively (Mohamad Za’ba & Kamarul Bahrin 2024; Truter & Fouché 2019; Truter, Theron & Fouché 2014). Additionally, addressing factors that challenge their resilience is vital to enable them to thrive in their profession (Rose & Palattiyil 2020:39). Resilience is widely acknowledged as an adaptable emotional state influenced by an individual’s environment and the significant challenges they face (Collins 2016:4). It is not a permanent attribute but a skill that can be improved, offering several health benefits (Abur 2020). Resilience is a dynamic process associated with change over time that produces a preferred outcome for systems when faced with adversity (Ungar 2021). It stems from environmental factors that shape human development more profoundly than individual actions (Neenan 2018) and includes adaptive mechanisms and external influences that are crucial in managing significant stressors (Ungar 2021). Resilience skills and strategies are vital for managing work-related pressure in helping professions. To diminish the stigma associated with disclosing emotions and promote a culture of resilience, social workers should have platforms where they can openly share their feelings (Grant et al. 2014:885). Qualities such as confidence, self-awareness, independence, hopefulness, honour, positivity, passion and critical thinking support resilience and reduce stigmatisation (Grant & Kinman 2012).

Research highlights a gap in understanding resilience skills among social workers (Truter et al. 2014). Internationally, research by Abur (2020); Campbell, Taylor and McGlade (2017); Kears and McArdle (2012) and Stanley, Bhuvaneswari and Arumugan (2018) on resilience in social workers and child protection workers has indicated that the topic of resilience skills and strategies is inadequately covered. Abur (2020) also emphasises the importance of equipping social work students and professionals with resilience skills to manage personal and professional challenges. This is important as studies in several countries reveal high levels of trauma and exhaustion in the profession (Unison 2019), with resilience research focusing mainly on child protection workers, teachers, nurses, police and defence personnel (Aiken et al. 2012; Collins 2016; Mosson 2019; Neil & Kruger 2022; Truter et al. 2014; Van Breda 2023; Wabule 2020; Wood, Ntaote & Theron 2012). The growing focus on resilience among social workers in South Africa signals the need for further research to foster hope and empowerment within the profession (Abur 2020).

In light of the introductory remarks and the body of knowledge outlined earlier in the text, the researcher conducted a study in 2023 to explore the resilience skills and strategies of social workers to address this noticeable gap in the literature (Grant & Kinman 2020; Rose & Palattyil 2020; Truter et al. 2014). This research responds to one main research question: *What are the resilience skills and strategies of social workers?*

### Problem statement and rationale

Much research has been conducted to highlight the importance of resilience in the social work sector. However, research on the particular resilience skills and strategies South African social workers use to deal with the demands inherent in their line of work is still conspicuously lacking. The purpose of this study was to explore the complex network of resilience abilities and tactics that different social workers use to shield themselves from the negative effects of their line of work.

## Research methods and design

### Study design

The study used a qualitative approach with explorative, descriptive and contextual methods, employing semi-structured interviews to investigate social workers’ lived experiences in their natural environments (Merriam & Tisdell 2016:22).

### Setting

The study was conducted with field and intake social workers employed at the Department of Social Development (DSD) in Johannesburg. Participants were recruited from various service points within the Johannesburg Metro Region, including Pimville, Chiawelo, Senaoane, Midrand and the main regional office.

### Study population and sampling strategy

This study involved field and intake social workers from Johannesburg’s DSD. After ethical approval and authorisation from the DSD’s Research Administrator, the researcher collaborated with sub-office supervisors to inform eligible social workers about the study. One-on-one meetings introduced the project’s objectives, participants’ rights and benefits, allowing potential participants time to consider joining. Using purposive sampling, 13 social workers were selected based on data saturation, ensuring they provided valuable insights into resilience skills and strategies.

The inclusion criteria contributed to the focus and depth of the study. By selecting social workers with two or more years of experience, the research ensured that participants had enough exposure to workplace challenges, allowing them to form and apply resilience strategies. Limiting participants to those who understood key concepts like ‘resilience skills’ and ‘resilience strategies’ ensured the collection of relevant and meaningful data. Additionally, focusing on social workers from the DSD in Johannesburg provided context-specific insights into how resilience is built and applied within a particular organisational setting, ensuring the findings were both relevant and applicable. Participants who had rich information on resilience skills and strategies and an understanding of how to develop them were selected. The identification of participants with rich information on resilience skills and strategies was facilitated through pre-screening based on the inclusion criteria. This involved initial consultations with participants where they were asked to briefly describe their understanding of resilience skills and strategies, which further assisted in selecting those with meaningful insights.

Participants comprised black Africans aged 29 years–44 years, representing various ethnic groups (Venda, Sepedi, Zulu, Setswana and Xitsonga), recruited from different DSD service points within the Johannesburg Metro Region.

### Data collection

Data were gathered between October 2022 and January 2023 by the researcher, using semi-structured interviews, incorporating open-ended questions aimed at exploring social workers’ lived experiences concerning their resilience skills and strategies. For instance, one question posed was, ‘Can you describe the effectiveness of the resilience skills and strategies you currently use?’ This interview format provided the researcher with the flexibility to probe deeper through follow-up questions, allowing for richer and more detailed insights into participants’ experiences. Participants were initially recruited via email after gatekeeper and ethical consent was received. In this initial contact, the researcher outlined the study’s topic, purpose and participants’ rights, ensuring an understanding of voluntary involvement and contact information for the study supervisor and the Research Ethics Committee was provided for further inquiries. For those expressing interest, face-to-face introductory sessions were arranged to answer questions, discuss interview length and clarify the study’s benefits and responsibilities. A total of 13 participants who met the inclusion criteria agreed to participate after signing informed consent forms, with six potential participants declining because of time limitations or perceived lack of relevant knowledge. The interviews, lasting between 1 h and 1 h and 40 min, took place at locations convenient to both the researcher and participants, fostering comfort and trust. Data saturation was achieved by the 13th interview, where no new themes emerged upon review of the transcriptions. To confirm saturation, the researcher conducted peer reviews with the transcriber and supervisor, and triangulation was employed to ensure consistency across interviews and observations, reinforcing the fact that no additional data were necessary.

### Data analysis

Data analysis followed the eight-step process recommended by Tesch (Creswell 2014:196; Creswell & Poth 2018:183–184). Raw data were transcribed and coded according to emerging themes and categories. The researcher collaborated with an independent coder and the study supervisor during the data analysis phase to ensure accuracy and reliability in identifying themes and insights from the participants’ responses.

### Trustworthiness of the findings

The researcher adhered to the four elements of trustworthiness: credibility, transferability, dependability and confirmability (Burns, Grove & Gray 2013). Credibility was enhanced through member checking, where findings and themes were taken back to participants for validation. Transferability was supported by detailing limitations, including participant numbers, data-collection methods, session lengths and the data-collection timeframe. Dependability was deepened through the researcher keeping complete records of all phases of the research process – from problem formulation to data analysis decisions (Bryman, Bell & Teevan 2012:392). Confirmability was ensured by ensuring that personal values or theoretical biases have not unduly influenced the research process or findings (Bryman et al. 2012:392).

### Ethical considerations

Ethical clearance to conduct this study was obtained from the University of South Africa and College of Human Sciences Research Ethics Review Committee (Ref no: 58544747-CREC-CHS-2022). Following this clearance, gatekeeper permission from the DSD was secured on 10 June 2022, and permission from the supervisors of satellite offices was also obtained. Written informed consent was obtained from the participants of the study. Having granted permission, the researchers approached participants seeking informed consent to participate in the study. The informed consent, voluntary participation, management of information and debriefing were ethical obligations that were maintained. During the data-collection process, the participants were not exposed to maltreatment and were informed that they could terminate the interview whenever they felt uncomfortable (Creswell & Poth 2028:56). A professional counsellor was on hand to conduct debriefing sessions should the participants be affected during the interview.

## Results

This research identified the following three themes that will be elaborated on:

- Resilience skills and strategies employed by social workers to surmount challenges.
- Effectiveness of resilience skills and strategies employed by social workers.
- Social workers’ suggestions on what the department can do to maintain their resilience.

In ensuring discussion, each of these themes will be presented with various sub-themes.

### Theme 1: Resilience skills and strategies employed by social workers to surmount challenges

The participants shared their accounts of the difficulties they face and how these difficulties impact both their professional and personal lives. Subsequently, the researcher inquired about the resilience skills and strategies they employ to cope with these challenges. Some of the resilience strategies used by social workers include practicing self-care, receiving support from managers and colleagues, taking time off work and engaging in activities with family and friends. Additional strategies include exercising, sleeping well and maintaining boundaries between work and personal life (Calitz, Roux & Strydom 2014; Kheswa 2019; Tumwesinge 2021).

#### Sub-theme 1.1: Self-care

Participants emphasised self-care as a vital strategy to handle challenges within the DSD, viewing it as essential for managing work stress and sustaining resilience, rather than considering resignation. Some participants reported strengthening their resilience by focusing on positive aspects to lift their mood during tough times. Others noted prioritising tasks and delegating to social auxiliary workers to manage workload effectively. Participants shared these approaches to self-care:

‘I think practicing self-care is the best strategy for me … When things are not looking good at work, when I go home, I forget about it and focus on myself … spoiling myself is another strategy that I use … whenever I feel overwhelmed, I take a lunch break and go to the mall and buy myself something nice, or just do window shopping as a self-distraction.’ (Participant B, female, 38 years old)

‘When I am out of the office, I forget about work life and cheer myself up with what I like and people that I love. Self-care is very important to me because we live once.’ (Participant F, male, 38 years old)

#### Sub-theme 1.2: Separating work life and personal life

Participants highlighted the importance of maintaining clear boundaries between work and personal life:

‘I am that kind of a person who does not mix work and pleasure. Whatever happens at work stays there and what happens at home remains there.’ (Participant C, female, 42 years old)

‘I leave what happens at work there and what happens at home at home.’ (Participant H, male, 41 years old)

#### Sub-theme 1.3: Support from family and friends

The crucial role of family and friends is highlighted in the narratives shared by Participant I, Participant C and Participant M:

‘My family is one of my supporting structures. I have a loving family, and we love each other. We always have lunch and often go out and we support each other always.’ (Participant I, male, 44 years old)

‘[*T*]he skills and strategies that I usually use when I face challenges are talking to my friends. My friends understand and support me a lot. We share a lot of things, so it is easy for me to talk to them.’ (Participant M, female, 39 years old)

#### Sub-theme 1.4: Support from supervisors and colleagues

The significance of social support from supervisors and colleagues was highlighted by eight participants, as reflected in the following narratives:

‘I have a very supportive supervisor and very helpful one. Whenever I have a challenge, I know if I can share with her, she will make me feel good. Again, my colleagues are my pillar of strength. We normally share experiences on difficult cases, so I know when I am stressed, they are there for me. We are like a family, and we support each other.’ (Participant A, female, 36 years old)

‘[*A*]nd another strategy is to talk to my supervisor and colleagues because we normally support each other during challenging times, and we support each other a lot.’ (Participant H, male, 41 years old)

#### Sub-theme 1.5: Taking time off and going out with friends or family on holiday

Participants highlighted the fact that taking time off and going on a holiday with friends or family is an effective strategy for dealing with challenges:

‘Strategies like going out with my family on a vacation, talking to my sister and friends, going out shopping and spoiling myself, taking myself out for a spa treatment works for me when I am stressed.’ (Participant D, female, 38 years old)

‘I take myself out with friends or family because they are my pillar of strength. When I go out, I will laugh and do all the things that cheer me up, so it helps me to forget about everything else and at the end, I would feel relieved and be able to focus again.’ (Participant G, female, 29 years old)

#### Sub-theme 1.6: Intrinsic rewards and uplifting experiences

Participants emphasised the importance of intrinsic rewards and uplifting experiences:

‘[*I*]t gives me pleasure and satisfaction knowing that I have changed someone’s life. I enjoy seeing people happy with what I do, and, in that way, it gives me strength and motivation to do more.’ (Participant B, female, 38 years old)

‘… I also use positive thinking … I mean not all cases are unsuccessful, most of them become successful, so I encourage myself with them and in that way, I get the strength to move forward. There are clients whom I assisted, and they show appreciation. In that way, I motivate myself with such so to say not all is failing, at least I have done this and that.’ (Participant C, female, 42 years old)

### Theme 2: Effectiveness of resilience skills and strategies employed by social workers

Study participants confirmed the fact that resilience skills and strategies significantly aid them in overcoming work challenges, reducing stress and providing effective client services:

‘It works for me hey, whenever I speak to my colleagues, supervisor or meet with friends, I feel relieved. When I share my challenges with them, I know they will encourage me and also give me advice on how to deal with whatever hindrance I may be having. As I am saying, self-love is my best medication, when I take care of myself by going out, exercises, and spoiling myself, it gives me self-satisfaction. In fact, these skills and strategies do work for me, because when I apply them, I know I will feel energetic and able to find a way forward.’ (Participant B, female, 38 years old)

‘I think they do work for me because they help me a lot. Whenever I apply them, I feel relieved and able to focus more on my job. Again, it also assists me to view things from a different perspective. It also gives me emotional stability and also improves my job performance.’ (Participant H, male, 41 years old)

### Theme 3: Social workers’ suggestions on what the department can do to maintain their resilience

Participants’ suggestions on what can be done to support social workers’ resilience will be presented next. The social workers’ suggestions varied, and they are covered under the following five sub-themes.

#### Sub-theme 3.1: Management Support and Consultation

Participants underscored the importance of management support in enhancing social workers’ resilience, aligning with the findings of Naples (2014) that organisations have both moral and legal responsibilities to safeguard their employees’ well-being:

‘I think if the Department can give its employees the necessary attention, it would be helpful. By necessary attention, I mean support and making it a point that they visit our offices regularly so that they can witness and be familiar with the kinds of workplace we are working in.’ (Participant A, female, 36 years old)

‘[*I*]t will be best if the employer can engage employees from the grassroot level on the decision-making. They must also provide employees with necessary support that they need. They must have meetings with employees which do not necessarily concern work-related issues but the well-being of employees. The employers must also emphasise with the employees and understand what they are going through so that the employees must also feel appreciated, and, in that way, it will assist them to perform their job effectively.’ (Participant H, male, 41 years old)

#### Sub-theme 3.2: Addressing high caseloads and employing more social workers

Participants highlighted the need to address high caseloads and employ more social workers to enhance their resilience:

‘The issue of caseloads should be addressed as it causes stress to employees. You cannot, as a social worker, have more than sixty cases, because clients end up suffering, children abused without our knowledge because we see them once per year because there are many. Now if one can have a manageable caseload, it will be easier to render supervision to each of them on a quarterly basis.’ (Participant G, female, 29 years old)

#### Sub-theme 3.3: Provide needed resources

Participants recommended that the department provide the necessary resources to carry out their duties efficiently and stressed the significance of standard procedures to support social workers’ personal safety and maintain client confidentiality:

‘I think the Department must improve regarding resources. If they can provide enough resources, it can assist us employees to thrive in our job. It will also assist employees to perform their job effectively.’ (Participant I, male, 44 years old)

#### Sub-theme 3.4: Reinstate performance bonuses and increase salaries

Participants suggested that the department should reinstate performance bonuses and increase social workers’ salaries, as it would serve as a motivating factor to work harder and boost their resilience:

‘[*T*]he Department must learn to acknowledge the good work done by the employees. So, I suggest that they can at least try to re-look at the issue of performance bonuses. Employees get encouraged when they get something to show appreciation at the end of the year. The little incentives that they used to give us used to motivate us …’ (Participant H, male, 41 years old)

#### Sub-theme 3.5: Publicise and improve access to the employee assistance programme

Participants recommended that the existence of employee assistance programme (EAP) should be widely publicised, and its services made easily accessible. They emphasised that the visibility of the EAP is essential for social workers to seek counselling and support, ultimately enhancing their resilience skills and strategies:

‘I heard our department has an Employee Assistance Programmes around Johannesburg. I have never seen any official from their offices, or even heard any colleague consulting their offices. So, the Department must make us aware of these offices and their officials so that we know where to go when we face challenges. Let them avail themselves, and make us aware of the services they render, because we are really going through stuff as workers, and we end up being hospitalised or go to private institutions when we need help while we have our own in the Department.’ (Participant G, female, 29 years old)

## Discussion

The findings of this study illustrate the challenges social workers face in their profession and the importance of employing resilience skills and strategies to cope with these challenges. Social work is inherently demanding because of the emotional strains of the profession (Grant & Kinman 2020; Rose 2021; Travis et al. 2016). Social workers experience various challenges while maintaining their professional confidence and personal well-being. These challenges include a lack of support, insufficient resources, high caseloads and client-related issues (Mashego 2018; Moyane 2016; Stevenson 2016; Truter & Fouché 2019). The adverse impact of these challenges affects social workers both professionally and personally. An unconducive working environment negatively influences their emotions, job performance, relationships with colleagues and social lives (Hardy 2017).

To cope effectively, it is essential for social workers to develop and utilise resilience skills and strategies. These strategies enable them to adapt and recover from the difficulties they face in their work. The ecological systems theory supports this, positing that an individual can be better understood in relation to their environment (Ahmed, Amal & Kilawin 2017; Guy-Evans 2020). This theory enables a comprehensive account of all systems and their connections, focusing on adjustment, stress and person–environment fit, crucial for resilience development (Van Rensburg, Theron & Rothman 2015). Resilience, while partially an inherent trait, is also shaped by systemic factors and can be enhanced through targeted interventions (Ungar 2021:42). Given this dual nature, social workers benefit from actively developing resilience skills, which are essential for navigating the complex challenges of their work environment effectively (Hardy 2017).

The resilience skills and strategies employed by social workers to overcome challenges include a range of self-care approaches, boundary-setting practices and sources of support, all of which play a vital role in fostering resilience within their challenging profession. The findings indicate that self-care, a central resilience strategy, enables social workers to maintain a balance between their demanding professional lives and personal well-being. Grant and Kinman (2012) support the view that self-care is fundamental to resilience, highlighting its role in sustaining mental health. These strategies involve cultivating life skills and self-control techniques, such as stress and anger management, which allow social workers to foster self-compassion and navigate work challenges effectively (Ostadhashemi et al. 2019; Grant & Kinman 2020).

In maintaining resilience, social workers also emphasise the importance of separating work from personal life. Clear boundaries, as noted by Salloum et al. (2015), are essential for sustaining well-being. Family and friends serve as critical support systems, grounding resilience within social relationships and networks rather than solely within individual qualities. Van Breda (2018) and Ungar (2021) underscore the influence of familial and social connections on resilience, illustrating the fact that supportive relationships provide resources that help social workers endure challenges. Theron (2020) further emphasises the fact that resilience is co-created by individuals and their social environments, reinforcing the value of relational dynamics.

Support from supervisors and colleagues is equally pivotal. Narratives affirm the importance of social support in alleviating workplace stress, a sentiment echoed by Kheswa (2019), who asserts that supervisor and peer support can help reduce the pressure social workers face. Peer supervision, as Ingram (2013) notes, fosters an informal yet essential support network that strengthens resilience.

Additionally, social workers highlight the importance of taking time off and spending leisure time with loved ones. Such activities are critical for achieving work–life balance, a necessity for long-term resilience, as confirmed by Salloum et al. (2015). Beyond these practices, intrinsic rewards play a role in uplifting social workers, with the satisfaction derived from making a positive impact on clients’ lives serving as a source of strength and motivation. Tumwesigye (2021) supports this, noting that job satisfaction can enhance morale and resilience. These strategies are essential for preserving social workers’ commitment to their profession, helping them maintain their well-being and productivity in a challenging work environment.

Resilience skills and strategies are essential for social workers, playing a key role in managing stress and maintaining service quality. Grant and Kinman (2020) highlight the fact that these skills empower social workers to handle stress effectively and uphold high care standards. Resilience strategies help them draw on personal strengths, avoid negative thought patterns and respond flexibly and adaptively to professional challenges. Self-care practices, particularly physical activities such as exercise, emerged as effective strategies for boosting confidence, positivity and problem-solving abilities. Cuddy (2015) supports this, highlighting the connection between body and mind practices in building personal power and confidence. The research findings indicate that resilience skills instil confidence and adaptability, equipping social workers with the tools needed to tackle both personal and work-related difficulties. Newman (2016) further highlights the fact that self-compassion enhances confidence and problem-solving capabilities, contributing positively to mental health and stress management.

The findings of this study also focus on the crucial role of departmental support and organisational resources in bolstering social workers’ resilience. Social workers report that management support and consultation are essential factors in helping them navigate workplace challenges. This aligns with Ungar’s (2014) concept of social connectedness as a protective dimension of resilience within social-ecological systems, where stable, trust-based relationships foster resilience. The study reveals that social workers view supportive relationships with colleagues, supervisors and managers as fundamental to their ability to cope with the demands of their roles, echoing Truter et al.’s (2014) findings that peer and management support enhances employees’ coping skills through consistent communication and debriefing.

High workloads, driven by staff shortages and a growing number of social issues, emerge as key stressors for participants. This finding is supported by Moyane (2016), who highlight that workload pressures in social work are exacerbated by limited resources and the rising complexity of social problems. The study indicates that reducing caseloads would allow social workers to manage stress more effectively, which is crucial for maintaining resilience and well-being.

The study also highlights the importance of access to resources and improved working conditions. Participants stress that a lack of adequate resources hampers their resilience strategies. This is supported by the IFSW (2012), which underscores employers’ responsibility to ensure safe and supportive work environments for social workers. Resources are essential for resilience, as noted by Day et al. (2015), who observe that access to adequate resources contributes significantly to employees’ overall well-being and performance.

Another finding is the role of financial incentives, such as performance bonuses and salary increases, in enhancing resilience. Social workers note that inadequate compensation contributes to stress and impacts their job satisfaction, aligning with findings by Calitz et al. (2014), who associate low salaries with burnout and stress among employees.

Finally, the study points to the need for greater awareness and accessibility of EAPs. Social workers report that knowing where to seek support during work-related challenges is crucial for their resilience. Meyer et al. (2018) corroborate this, asserting the importance of EAP services in promoting employee well-being and providing support systems within the workplace.

### Recommendations

Based on participants’ suggestions, the following recommendations are proposed: Social workers are encouraged to engage in personal development, practicing resilience skills for both personal and professional well-being. Accredited Continuing Professional Development (CPD) providers and universities should integrate resilience training into curricula, focusing on emotional resilience, work–life balance and coping strategies. Additionally, the Department should promote a supportive work environment by establishing professional networks, regular debriefing sessions, counselling services and resources to help social workers manage job-related stress and build resilience.

### Limitations

The limitations of this study provide crucial insights into its scope and applicability, highlighting potential constraints and biases that may have influenced the research outcomes, thereby safeguarding its credibility. Key limitations include the demographic composition of participants, which was primarily black social workers with a gender imbalance of nine females and four males, potentially not fully representing diverse perspectives. The geographic focus on Johannesburg offices of the DSD restricts generalisability to other regions or non-governmental organisations. Additionally, the qualitative research approach, while offering in-depth insights, limits the ability to generalise findings beyond the specific context of this study.

## Conclusion

This article provided narratives illustrating the importance of resilience skills and strategies employed by social workers to address their professional challenges. The types of challenges they encounter and the resilience skills and strategies they utilise to manage these difficulties were thoroughly explored. Additionally, participants offered suggestions on how their employers could support their resilience. The article emphasised the responsibility of the DSD, the practice and social workers themselves in ensuring the well-being of social workers by providing the necessary support. The resilience skills and strategies highlighted in this study are essential for maintaining the well-being and professional effectiveness of social workers.
